# Ecthyma Gangrenosum-Like Lesion in a Patient With a Leukocyte Adhesion Defect: Pseudomonas aeruginosa, Staphylococcus aureus, and a Rare Agent, Fusarium

**DOI:** 10.7759/cureus.93580

**Published:** 2025-09-30

**Authors:** Fatma Tuğba Çetin, Ümmühan Çay, Asena Ünal, Arbil Açıkalın, Özlem Özgür Gündeşlioğlu, Dilek Özcan, Mahir Serbes, Derya Alabaz

**Affiliations:** 1 Pediatric Infectious Diseases, Cukurova University Balcalı Hospital, Adana, TUR; 2 Pathology, Cukurova University Balcalı Hospital, Adana, TUR; 3 Pediatric Immunology and Allergic Diseases, Cukurova University Balcalı Hospital, Adana, TUR

**Keywords:** child, ecthyma gangrenosum, fusarium, leukocyte adhesion defect, skin infection

## Abstract

*Fusarium *species can cause serious infections, including superficial and invasive ones. In immunocompromised individuals, they can cause skin infections such as ecthyma gangrenosum and disseminated disease through lymphatic spread. In this case report, we present a nine-year-old girl with ecthyma gangrenosum whose primary disease was a leukocyte adhesion defect (LAD). She developed a burn on her right lower extremity after spilling hot tea. *Pseudomonas aeruginosa, Staphylococcus aureus,* and *Fusarium *spp*.* were isolated from a wound culture taken from her ankle. While *Pseudomonas aeruginosa* is generally the causative agent of ecthyma gangrenosum, *Fusarium *species can also be encountered in patients with neutropenia or neutrophil dysfunction. It should be noted that *Fusarium *species, a rare mold pathogen, can also cause serious skin infections in immunocompromised patients, particularly after burns.

## Introduction

*Fusarium*, a saprophytic fungus, is widely found in soil [1]. After* Aspergillus*, it is the second most common mold infecting humans [2]. In recent years, the incidence of *Fusarium*, an opportunistic pathogen that causes invasive or localized infections in humans, has increased [3,4]. While *Fusarium* is sporadically seen in immunodeficiencies such as chronic granulomatous disease and leukocyte adhesion defect (LAD), its incidence is increasing in patients undergoing bone marrow transplantation. In a hospital in the United States, the incidence of *Fusarium* infection over a 10-year period was 1.2% in 750 allogeneic bone marrow transplant recipients and 0.2% in 1537 autologous bone marrow transplant recipients [5]. A study examining *Fusarium* infections in acute leukemia patients in Italy found the incidence to be 0.06% [6].

*Fusarium* usually presents as superficial lesions, such as onychomycosis and keratitis, in immunocompromised patients. It can spread rapidly via lymphatic spread in patients with neutropenia, neutrophil dysfunction, immune deficiency, organ transplant recipients, and malignancies, causing serious infections such as ecthyma gangrenosum [1,7]. Furthermore, *Fusarium* has been reported to cause sinusitis, peritonitis, thrombophlebitis, and osteomyelitis, although rarely [8]. Early diagnosis and appropriate treatment of *Fusarium* infections in patients with risk factors are crucial for reducing mortality. If left untreated, it is associated with high mortality and has an aggressive course [2].

In this case report, we present a nine-year-old female patient with ecthyma gangrenosum, whose primary disease was LAD. She sustained burns on her lower extremity due to a hot tea spillage. *Pseudomonas aeruginosa (P. aeruginosa), Staphylococcus aureus (S. aureus),* and *Fusarium *spp*. *were isolated from the wound culture taken from her ankle.

This article was presented as an abstract at the 18th National Congress of Pediatric Infection and Immunization on February 21, 2025.

## Case presentation

**Figure 1:**
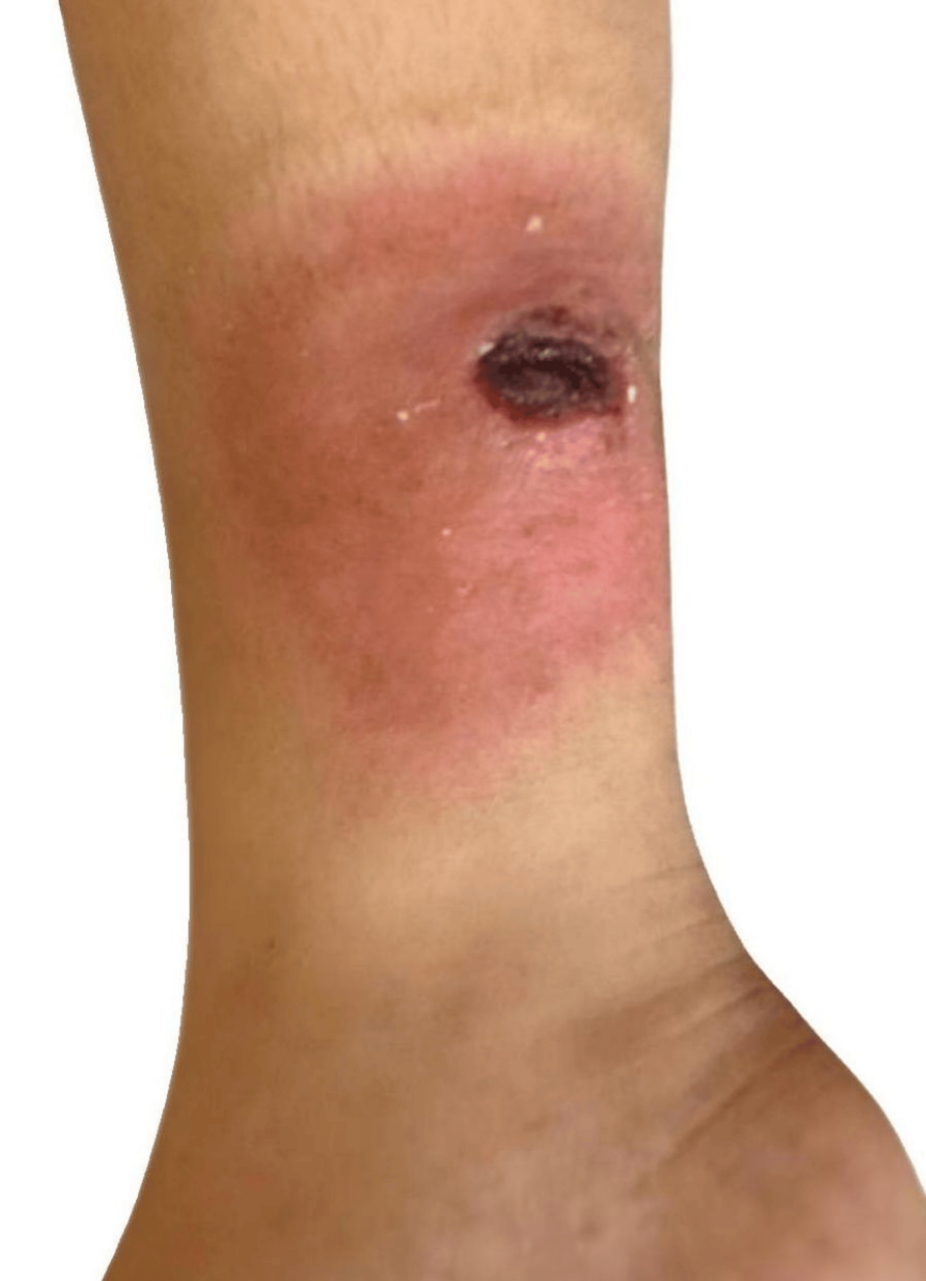
A 3x4 cm open wound on the medial aspect of the right leg, necrotic due to a burn, and a surrounding hyperemic area of approximately 5 cm.

A nine-year-old female patient with LAD was reported to have spilled hot tea on her right leg at home five days prior. She was admitted to the outpatient clinic due to a right ankle wound. On initial examination, a 3x4 cm burn-related necrotic open wound with a surrounding 5 cm hyperemic area (Figure 1) was detected on the medial aspect of her right leg, and the patient was consulted with us.

Physical examination revealed no pathological findings other than ecthyma gangrenosum. Her temperature was 36.5°C, her blood pressure was 100/75 mmHg, and her pulse was 80 beats per minute. Leukocytes were 20,000 μL, neutrophils were 13,800 μL, Hgb was 11.1 g/dL, platelets were 406,000 μL, C-reactive protein was 17.06 mg/L (reference range 0-5 mg/L), and liver and kidney function tests were normal. Superficial ultrasonography revealed marked thickening and edematous skin and subcutaneous tissue on the medial aspect of the right leg, along with cellulitis. Given the patient's primary disease and clinical presentation, hospitalization was recommended.

**Figure 2:**
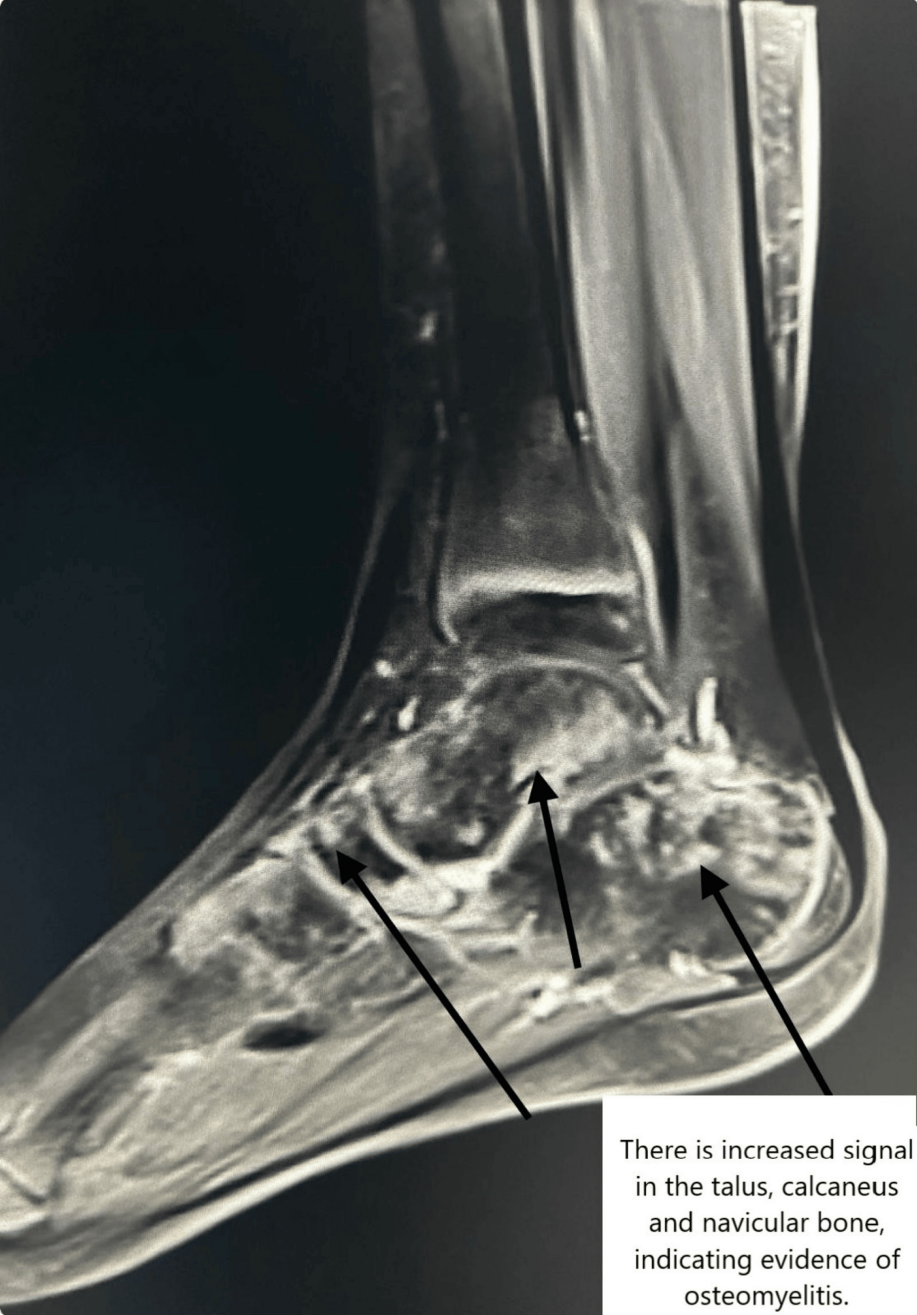
Magnetic resonance imaging revealed increased signal in the talus, calcaneus, and navicular bones of the right foot, suggesting osteomyelitis (arrows).

Empirical meropenem and teicoplanin were started after culture samples were obtained. *P. aeruginosa* (meropenem sensitive) and *S. aureus* grew in the first wound culture. There was no growth in the blood culture. Fluconazole was added to the treatment of the patient, who did not respond to it, and a skin biopsy was performed. Magnetic resonance imaging (MRI) was performed for osteomyelitis. The signal increase was observed in the talus, calcaneus, navicular, and cuboid bones of the right foot, and the findings were evaluated in favor of osteomyelitis (Figure 2).

**Figure 3:**
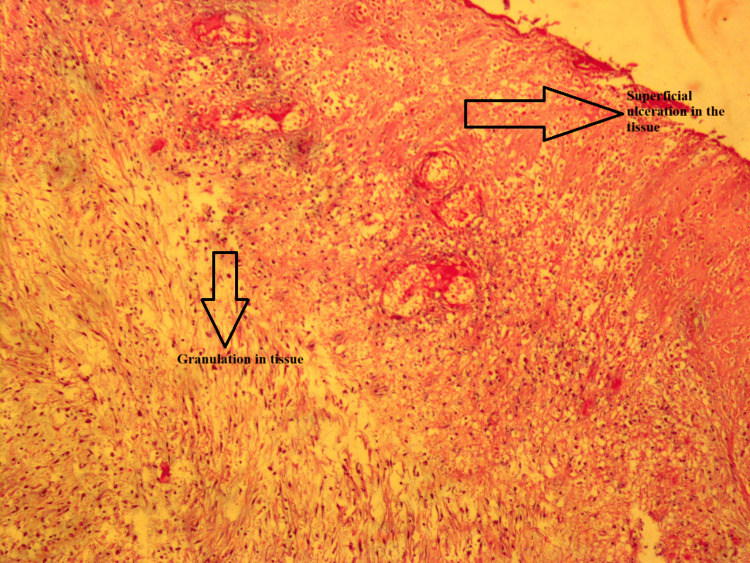
Active chronic ulcerative inflammation (hematoxylin-eosin x100).

Skin biopsy revealed 'Active chronic ulcerative inflammation, granulation tissue, pyoderma gangrenosum?' (Figure 3). A steroid was added to the treatment for pyoderma gangrenosum. Donor granulocyte transfusion (DGT) was also administered.

**Figure 4:**
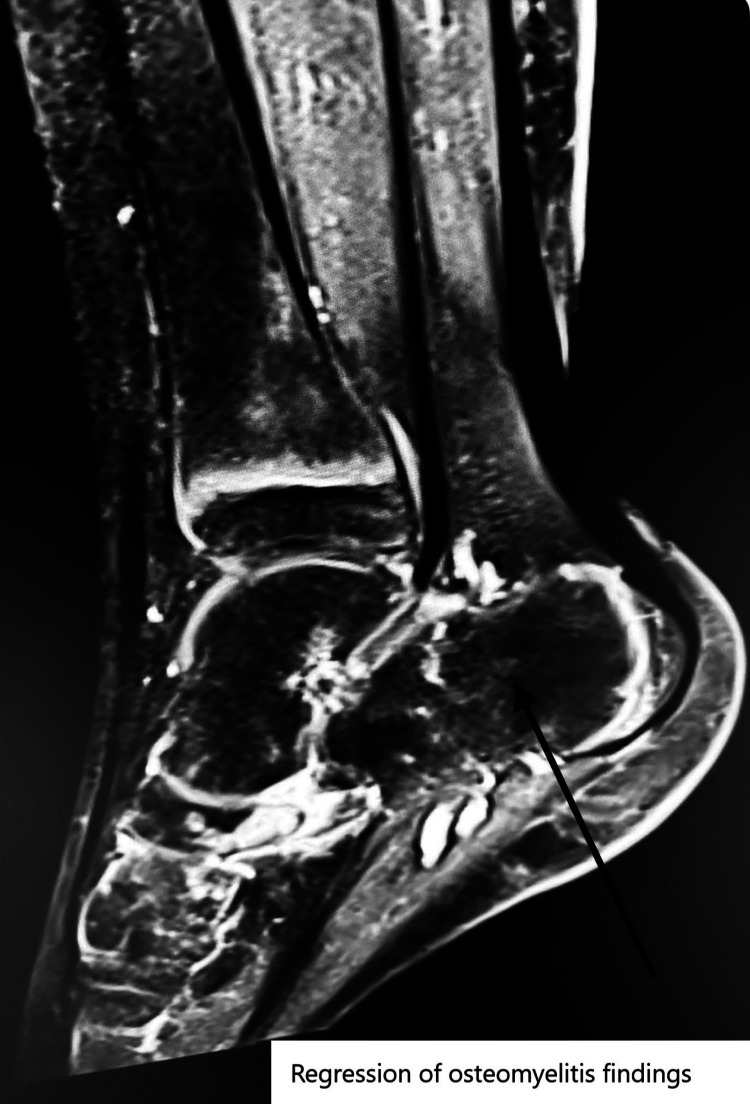
Regressive osteomyelitis findings on magnetic resonance imaging (arrow).

Although osteomyelitis findings completely regressed on follow-up MRI (Figure 4), wound healing did not occur. 

**Figure 5:**
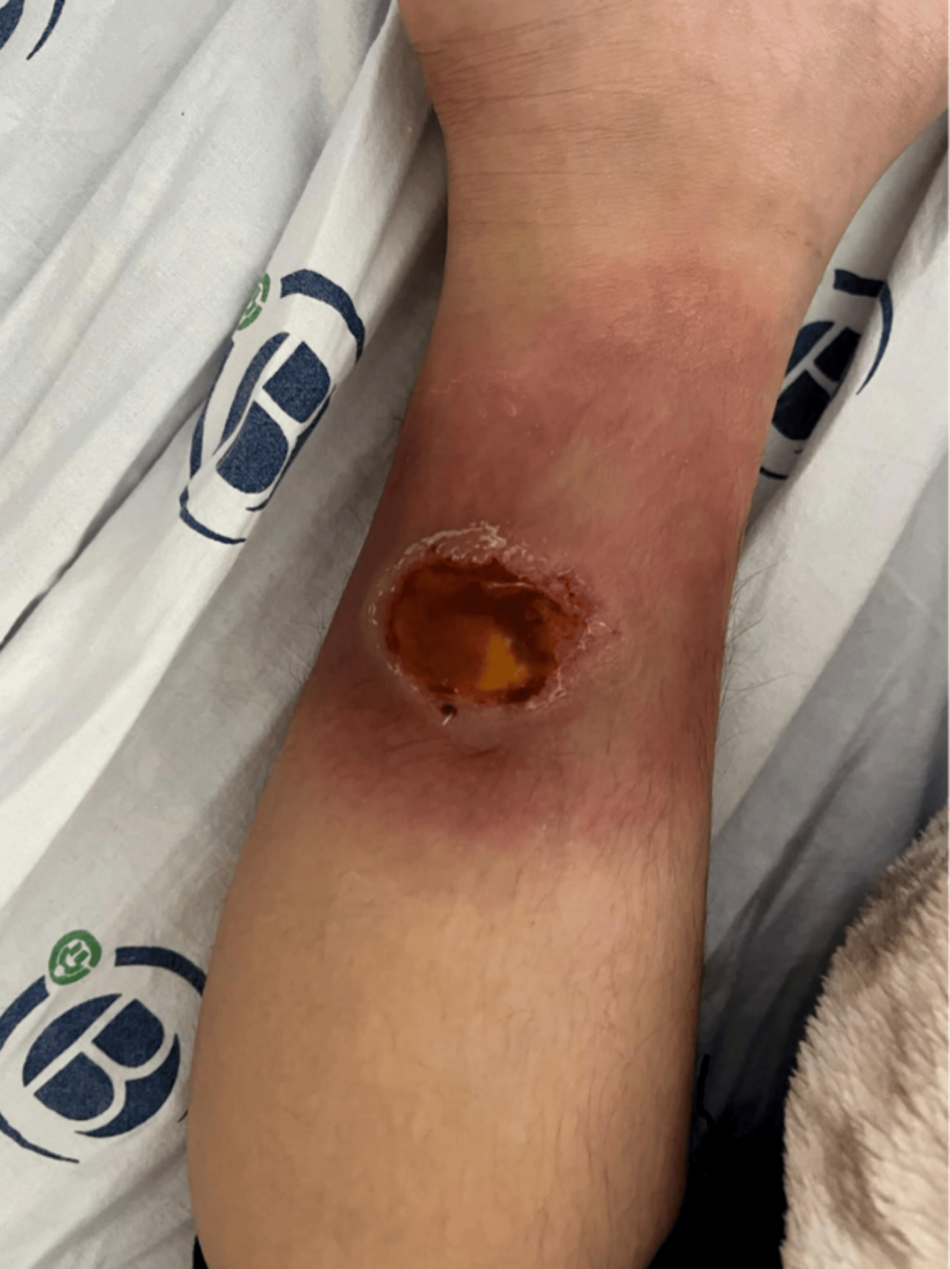
A 5x6 cm non-healing wound with hyperemia extending almost to the entire ankle and a crusted, necrotic area.

Figure 5 shows the patient's 5x6 cm non-healing wound, which includes hyperemia and a crusted, necrotic area extending almost to the entire ankle.

**Figure 6:**
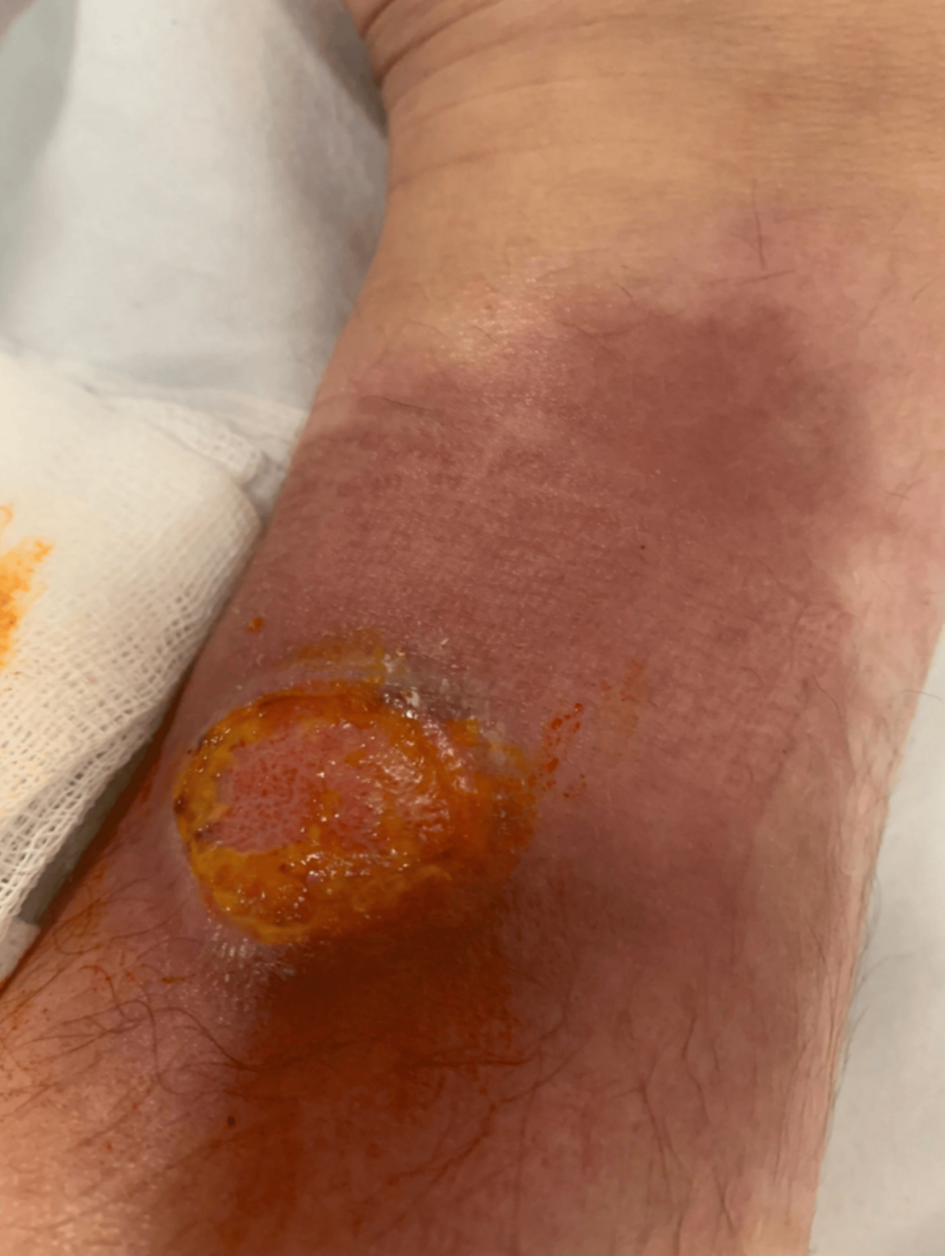
A 3x3 cm wound with a diminishing area of hyperemia surrounding it, healing necrotic tissue, and new tissue formation.

Tissue culture was taken from the patient again and sent to the Public Health Laboratory. Fluconazole was stopped, and voriconazole was started, considering rare mold fungi in the treatment of the patient. The *Fusarium* culture sent to the Public Health Laboratory was positive. She received intravenous voriconazole treatment for *Fusarium* for 23 days. In the follow-up, the wound site was better, and the patient was discharged with oral voriconazole and trimethoprim/sulfamethoxazole treatment. Figure 6 shows that the patient's wound has shrunk (reduced to 3x3 cm). Additionally, the area of hyperemia surrounding it has diminished, necrotic tissue has healed, and new tissue formation is evident.

**Figure 7:**
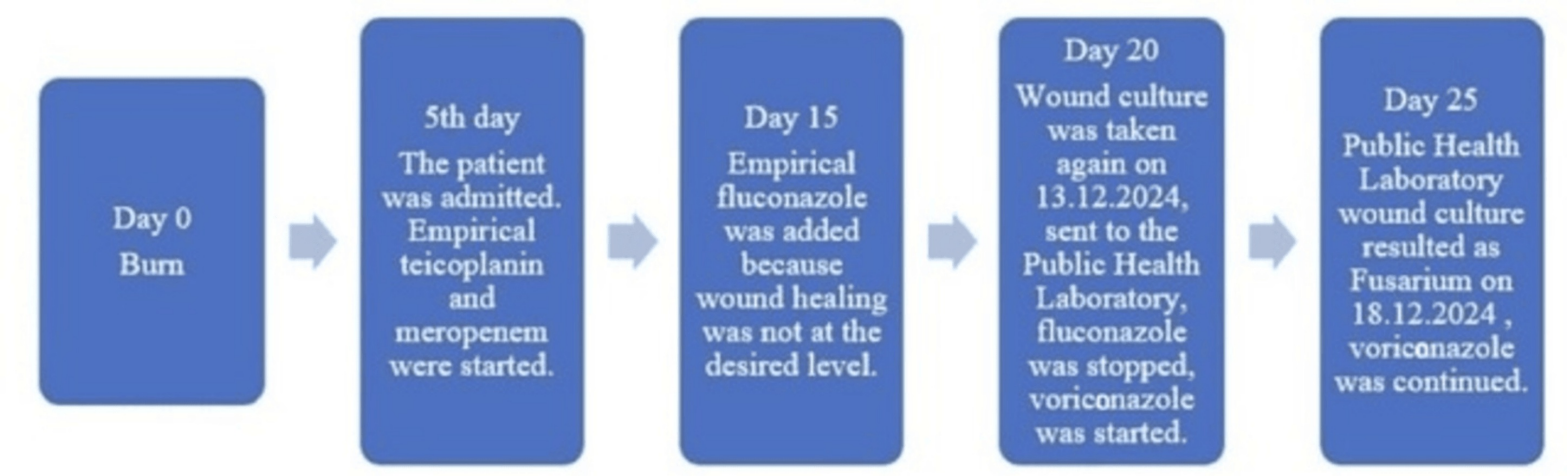
Patient flow chart.

The flow chart from the patient's burn injury to the present day is shown in Figure 7. The patient has been closely monitored for eight months. We planned to complete voriconazole treatment in 12 months. She is also being followed in the immunology and dermatology departments.

## Discussion

Ecthyma gangrenosum is a rare dermatologic infection that is typically caused by bacteria such as *P. aeruginosa, Staphylococcus,* and *Streptococcus*. It is more prevalent in patients with malignancy and immunodeficiency [9]. In the literature, *Fusarium *species have also been identified as the causative agent of ecthyma gangrenosum, albeit less frequently [3,10].

Skin lesions caused by *Fusarium *spp. can be localized or diffuse. Localized lesions are usually seen as cellulitis. Patients have a history of trauma or onychomycosis. In our case, hot tea was spilled on her feet, and the initial clinical picture was consistent with cellulitis. Skin infection may develop into a disseminated form. Multiple erythematous papular or nodular painful lesions typically develop in cases with disseminated disease [10,11]. Necrosis, which frequently occurs in the center of the lesions, gives the lesion an ecthyma gangrenosum-like appearance, as seen in our case.

Our patient with LAD had *P. aeruginosa* and *Fusarium *spp*.* growth in wound cultures taken at different times. In the study by Mellouli et al. in the literature, it was observed that a nine-year-old patient with LAD developed a knee abscess as a result of a fall, and *P. aeruginosa* and* Fusarium solani *were grown in blood and abscess cultures. This case was treated with DGT and targeted antibiotic and antifungal therapy for more than 1.5 years and recovered [10]. Our case had no growth in the blood culture. In a study conducted in our country, ecthyma gangrenosum-like skin lesions caused by disseminated *Fusarium* infection were found in a 52-year-old patient with acute myeloid leukemia. *Fusarium *growth was also observed in the skin biopsy culture of this case [11].

The prognosis of *Fusarium* infections is directly related to the immune status of immunocompromised individuals. Because of the high mortality rate of the disease, a biopsy should be taken from the lesion immediately in case of any suspicion, and treatment should be started [9]. In our case, a biopsy was performed early, and findings consistent with pyoderma gangrenosum were detected; prednisolone was added to the treatment. DGT was performed once or twice weekly to enhance the immune response. Pyoderma gangrenosum can also be associated with other diseases associated with immune system dysfunction, and the lack of consensus on diagnostic criteria makes the differential diagnosis critical. It is important to consider malignancy, infection, or cutaneous vasculitis in the differential diagnosis. Most importantly, pyoderma gangrenosum can coexist with these conditions in some cases [12,13]. In our case, the pathology result was consistent with pyoderma gangrenosum, and* Fusarium* was grown in the wound culture.

First-line treatment for *Fusarium *infections is voriconazole and amphotericin B, with posaconazole as salvage therapy [14]. The most important factors determining recovery and survival are recovery of neutrophil function and resolution of neutropenia [2]. Immunology and dermatology should also be monitored for pyoderma gangrenosum and ecthyma gangrenosum.

## Conclusions

Although *P. aeruginosa* is usually the causative agent of ecthyma gangrenosum,* Fusarium* species can also be a causative agent in neutropenic patients or those with neutrophil dysfunction. In this patient with LAD, wound cultures taken at different times after burns yielded *P. aeruginosa* and *Fusarium *species. In cases that fail to respond to *P. aeruginosa* treatment, it should be kept in mind that *Fusarium *species, a rare mold, can cause serious skin infections in immunocompromised patients. We believe this rare case will shed light on the path of physicians.
